# Differential impact of working hours on unmet medical needs by income level: a longitudinal study of Korean workers

**DOI:** 10.5271/sjweh.3999

**Published:** 2022-02-25

**Authors:** Dong-Wook Lee, Jaesung Choi, Hyoung-Ryoul Kim, Jun-Pyo Myong, Mo-Yeol Kang

**Affiliations:** 1 Public Healthcare Center, Seoul National University Hospital, Seoul, Republic of Korea; 2 Department of Economics, Sungkyunkwan University, Seoul, Republic of Korea; 3 Department of Occupational and Environmental Medicine, Seoul St. Mary’s Hospital, College of Medicine, The Catholic University of Korea, Seoul, Republic of Korea

**Keywords:** health inequity, income level, unmet medical need, working hour

## Abstract

**Objectives:**

Unmet medical need is defined as the perceived need for medical service that is not received. Although the association between unmet medical needs and working hours has been explored before, the combined effect of household income has not been investigated thus far. This study, therefore, aimed to examine the differential association between working hours and the risk of unmet medical needs according to household income.

**Methods:**

A total of 7047 participants enrolled in the Korea Health Panel data 2011–2014 were considered. The analytical method used in this study was a generalized estimating equation model that accounted for repeated measured participants. By controlling for time-invariant individual-fixed effects, we identified the relationship between long working hours and the risk of unmet medical needs.

**Results:**

The association between long working hours and the risk of unmet medical needs differed according to household income. In the highest quintile of household income, the risk of unmet medical needs was 1.58-fold higher among those who worked >52 hours per week than among those who worked 30–52 hours per week. However, this association was not significant in the lowest quintile group.

**Conclusions:**

The current study implies that financial hardship might be a more fundamental health hazard than working longer hours among the low-income group. Future policies should consider not only limiting working hours but also compensating workers’ income to adequately protect low-income workers from the health risks associated with long working hours.

Unmet medical need is defined as the perceived need for a medical service that is not received (1), suggesting that it is not possible to receive treatment despite the desire for medical services. Studies show that unmet medical needs can lead to a number of adverse consequences, including increased morbidity, more severe disability (2), increased emergency room visits (3) and acute care hospitalization (4), psychological distress (5), poor self-assessment of health and quality of life, and increased mortality (6). Hence, it is considered an important variable to evaluate equal access according to need (7).

The 2016 Korean National Health and Nutrition Examination Survey (KNHANES) reported that approximately 8.8% of the general population in Korea had experienced unmet medical needs at least once a year (8); in contrast, among the 28 countries of the European Union, an average of 2.5% of the total population had experienced unmet medical care in the same year (9). The scope of medical services has expanded to include psychological and social needs of patients to support their emotional and psychological well-being; consequently, rather than focusing on the existing treatment-oriented service provision (10), access to and the use of necessary medical services has become essential to address health problems and secure a good quality of life (11). Therefore, it is important to identify unmet medical needs and understand the reasons for the same, by integrating various perspectives on patients’ medical needs (11).

According to prior studies, the factors that drive unmet medical needs can be categorized into availability, accessibility, and acceptability (7). Availability emerges when the required services have not been provided at the desired time or place. Accessibility includes difficulties in accessing medical services owing to financial constraints that hamper the ability to pay, for instance, for medical expenses or transportation. Acceptability occurs because patients lack time to accept services, do not perceive the need for medical services, or have low acceptance of attitudes and knowledge, such as a lack of information about service providers.

Among the various factors that influence unmet medical needs, a lack of time owing to long working hours could cause difficulties in availability and acceptability. The adverse effects of working hours on health have been a major concern for the well-being of workers. Despite the large body of research on the association between long working hours and various health problems (12), few studies have specifically investigated the association between unmet medical needs and long working hours. Seok et al (13) analyzed data from the KNHANES, collected during 2007–2012, which included 8369 participants (4765 males, 3604 females) aged 20–54, who were paid workers (13). The results showed that there was a dose–response relationship between weekly working hours and unmet healthcare needs; however, the cause of unmet healthcare needs and its association with income status were not considered. Consequently, additional research on this topic is required using longitudinal data.

Meanwhile, low-income is a well-known socio­economic factor leading to an increased risk of unmet medical needs (7). Theoretically, with all other factors being controlled, longer working hours generally accompany higher individual incomes. However, in reality, individuals may want to work fewer hours if their wages are sufficient to afford a decent standard of living for household members, as depicted in a backward-bending labor supply curve (14). In contrast, low-income workers need to work longer hours to secure a sufficient living income. From a labor economics perspective, individuals allocate their time between work and leisure to maximize their utility, given budget and time constraints (15). Accordingly, working hours may have different effects on workers’ health depending on their economic background. A study using nationally representative data from South Korea found a significant association between long working hours and estimated cardiovascular disease risk among high-income participants, and no such association in low-income participants (16).

Although the association between unmet medical needs and working hours has been suggested before, to the best of our knowledge, epidemiological evidence with longitudinal data has not been reported. More importantly, the combined effect of household income has not been investigated. Therefore, this study aimed to examine the differential association between working hours and the risk of unmet medical needs according to household income.

## Methods

### Study population

This study used the Korea Health Panel (KHP) data collected by the Korea Institute for Health and Social Affairs and the National Health Insurance Service. The KHP is a national survey constructed through stratified, multi-stage cluster sampling from the population census data of South Korea, and has been conducted annually since 2008. The primary purpose of the KHP is to provide data on the influence of individual, social, and environmental elements on healthcare expenditure (17). The KHP incorporates a wide range of information, including demographic and socioeconomic characteristics, health status (medical, physical, and psychosocial), working hours, income (pension, income, assets, and housing), and medical expenditure. All the survey items are repeated for each panel wave. Trained interviewers visit the study participants’ homes and conduct a computer-assisted personal interview. In the KHP data released from 2008 to 2018, information on the working hours of participants was collected only between 2011 and 2014. Therefore, we used the waves from the 2011 to 2014 KHP data of 23 212 individuals and 66 965 observations. Participants who were paid employees and aged 20–59 years, considering the official retirement age in South Korea, were included. Non-waged workers or non-workers [N=14 795 (63.7%)] and participants aged <20 or ≥60 [N=1176 (5.0%)] were excluded, as were those who had missing information on unmet medical needs [N=164 (0.7%)] and on working hours, household income, occupation, and shift work [N=30 (0.1%)]. Finally, 7047 participants were included in the current analysis (supplementary material, https://www.sjweh.fi/article/3999, figure S1). The distribution of gender, age, employment status, and household income are presented in supplementary table S1.

### Variable measurements

Participants’ weekly working hours were recorded using the following questionnaire item: “How many hours do you work per week, including overtime?” Considering South Korea’s labor laws, participants’ weekly working hours were classified into three groups: ≤30 (short weekly working hours), 30–52 (standard, and the most frequent weekly working hours) and > 52 hours (overtime work allowed in extraordinary situations).

Unmet medical needs were measured by the subjective judgement of the need for medical services. When a participant could not use medical services for various reasons, despite having a medical need, they were judged to have an unmet medical need. Accordingly, participants were asked, “Did you have an experience in the past year in which you were not able to receive the medical service you needed?” The answer “yes” was considered as a case of unmet medical need. If a participant did not receive the necessary medical services, they were asked: “If you could not receive a medical service when you needed it, what was the reason for that?” Respondents could choose between the following options: “For economic reasons”, “I did not have time to visit”, “Because the medical institution is far away”, “Difficult to visit owing to mobility or health reasons”, “Because there was no one to take care of the child”, “Because the condition is mild”, “Do not know where to go”, “Because it could not be booked in a short time”, and “I did not have a family doctor”. Additional analysis was performed on “economic reasons” and “lack of time”, which received the highest number of responses.

In this study, age and gender were used as demographic characteristics, and household income as socioeconomic variables. Age was grouped into 20–29, 30–39, 40–49 and 50–59 years. Household income, including labor and financial income, was calculated on an equalized scale by dividing income by the square root of the number of household members (18); further, it was categorized into quintiles from lowest income (Q1) to highest income (Q5) among all survey participants. Job classes were gathered using the Korean Standard Classification of Occupation. We classified managers, professionals and related workers, and office workers into white-collar and service workers, sales workers, skilled agricultural, forestry and fishery workers, craft and related trade workers, equipment, machine operating and assembling workers, and elementary workers as blue-collar workers. Depending on their smoking status, as assessed by questionnaire items, participants were classified into current, former, and non-smokers. Shift work, associated with increased unmet healthcare need (19), was assessed using the following questionnaire item: “Do you usually work during the daytime between 06:00 and 18:00, or in other schedules?”

### Statistical analysis

The general characteristics of the study population and the prevalence of unmet medical needs were described. Compared to the reference group, where the weekly working hours were 30–52 hours, odds ratios (OR) and 95% confidence intervals (CI) for unmet medical needs of other weekly working hours groups (<30 and >52 hours) were calculated. We also calculated OR and CI for unmet medical needs of middle and low household income groups, compared with the highest household income group. The analytical method used in this study was a generalized estimating equation model that accounted for repeated measures. This model is suitable for multivariate analysis using time information by repeatedly measuring panel objects through a panel survey. The endogeneity problem, owing to unobservable individual and occupational characteristics, can be overcome using panel data (20). By controlling for time-invariant individual-fixed effects, we identified the relationship between long working hours and the risk of unmet medical needs. The covariates used in the model were age, gender, occupation group (blue- or white-collar), smoking status, shift work, and household income. The occupation group, shift work, and household income were time-varying variables for the repeatedly enrolled participants, according to the 2011 to 2014 waves of the KHP data. Additionally, the association between long working hours and unmet medical needs based on the reason was identified. Consequently, the data were classified into 15 groups according to 3 weekly working hours groups, the quintiles of household income group, and calculated OR, compared to groups with standard working hours (30–52 hours per week) and highest household income (5^th^ quintile). Sensitivity analyses were performed with different weekly working hours grouping (<30, 30–40, 41–52, and >52 hours). We used the PROC GENMOD protocol of the SAS version 9.4 (SAS Institute, Cary, NC, USA). The two-tailed P-values <0.05 were considered statistically significant.

## Results

Table 1 summarizes the participants’ general characteristics and prevalence of unmet medical needs by the year included in our analysis (2011–2014). The number of surveyed participants was 4071, 3812, 3591, and 4639 in 2011, 2012, 2013, and 2014, respectively. Males accounted for a greater number of participants than females (55.9%, 55.6%, 55.4% and 55.6% in 2011, 2012, 2013 and 2014, respectively). Most participants were in the age group of 40–49 (35.4%, 36.1%, 36.2%, and 35.7% in 2011, 2012, 2013, and 2014, respectively), and worked 30–52 hours per week (67.1%, 68.6%, 72.1%, and 73.6%, respectively). The mean weekly working hours in 2011–2014 were 46.5 [standard deviation (SD) 13.2], 45.5 (SD 12.7), 45.4 (SD 12.4), and 45.0 (SD 11.9), respectively. Supplementary table S2 shows the frequency of unmet medical needs. Throughout the study period, the proportion of participants with unmet medical needs was 16.5%, 14.7%, 16.4%, and 12.1% in 2011, 2012, 2013 and 2014, respectively. Those who worked >52 hours per week most commonly complained of unmet medical needs (21.6%, 17.9%, 18.3%, and 15.1% in 2011, 2012, 2013 and 2014, respectively). The most common reasons included lack of time and economic burden, which accounted for almost 50% and 20%, respectively.

**Table 1:** Demographic characteristics of the study participants. [SD=standard deviation].

	2011		2012		2013		2014	
			
	N (%)	Mean (SD)	N (%)	Mean (SD)	N (%)	Mean (SD)	N (%)	Mean (SD)
Gender								
Male	2216 (55.9)		2119 (55.6)		1966 (55.4)		2590 (55.6)	
Female	1749 (44.1)		1692 (44.4)		1585 (44.6)		2070 (44.4)	
Age (years)								
20–29	522 (13.2)		518 (13.6)		480 (13.5)		590 (12.7)	
30–39	1121 (28.3)		1027 (27)		921 (25.9)		1184 (25.4)	
40–49	1402 (35.4)		1374 (36.1)		1284 (36.2)		1664 (35.7)	
50–59	920 (23.2)		892 (23.4)		866 (24.4)		1222 (26.2)	
Weekly working hours		46.5 (13.2)		45.5 (12.7)		45.4 (12.4)		45.0 (11.9)
<30	253 (6.4)		272 (7.1)		218 (6.1)		295 (6.3)	
30–52	2662 (67.1)		2613 (68.6)		2561 (72.1)		3430 (73.6)	
>52	1050 (26.5)		926 (24.3)		772 (21.7)		935 (20.1)	
Occupation								
White-collar								
Manager	225 (5.7)		212 (5.6)		216 (6.1)		261 (5.6)	
Professionals and related workers	864 (21.8)		856 (22.5)		776 (21.9)		1042 (22.4)	
Office workers	621 (15.7)		599 (15.7)		586 (16.5)		820 (17.6)	
Blue-collar								
Service workers	375 (9.5)		357 (9.4)		303 (8.5)		439 (9.4)	
Sales workers	344 (8.7)		332 (8.7)		314 (8.8)		410 (8.8)	
Skilled agricultural, forestry and fishery workers	23 (0.6)		24 (0.6)		15 (0.4)		25 (0.5)	
Craft and related trade workers	508 (12.8)		503 (13.2)		476 (13.4)		581 (12.5)	
Equipment, machine operating and assembling workers	368 (9.3)		349 (9.2)		322 (9.1)		438 (9.4)	
Elementary workers	637 (16.1)		579 (15.2)		543 (15.3)		644 (13.8)	
Smoking								
Current smoker	1183 (29.8)		1111 (29.2)		1038 (29.2)		1390 (29.8)	
Former smoker	590 (14.9)		549 (14.4)		523 (14.7)		693 (14.9)	
Never smoker	2192 (55.3)		2151 (56.4)		1990 (56.0)		2577 (55.3)	
Shift work								
No	3560 (89.8)		3407 (89.4)		3182 (89.6)		4189 (89.9)	
Yes	405 (10.2)		404 (10.6)		368 (10.4)		471 (10.1)	
Household income (quintile)								
1^st^	144 (3.6)		136 (3.6)		129 (3.6)		141 (3.0)	
2^nd^	550 (13.9)		535 (14.0)		452 (12.7)		582 (12.5)	
3^rd^	901 (22.7)		845 (22.2)		786 (22.1)		1051 (22.6)	
4^th^	1109 (28.0)		1052 (27.6)		1036 (29.2)		1344 (28.8)	
5^th^	1261 (31.8)		1243 (32.6)		1148 (32.3)		1542 (33.1)	

The association between weekly working hours and unmet medical needs is presented in table 2. Unmet medical needs were significantly associated with weekly working hours. Compared with the 30–52 hours per week group, the >52 hours per week group showed a significantly higher OR (1.36, 95% CI 1.22–1.52) for unmet medical needs. Weekly working hours were also significantly associated with unmet medical needs owing to a lack of time and economic burden. Compared with the 30–52 hours per week group, the OR for unmet medical needs owing to lack of time, in the >52 hours per week group was 1.64 (95% CI 1.42–1.90). However, short work time (<30 hours/week) was significantly associated with a lower unmet medical need driven by a lack of time (OR 0.65, 95% CI 0.48–0.88), and a higher unmet medical needs from economic burden (OR 1.61, 95% CI 1.17–2.21). In the gender-stratified analyses, these findings did not differ notably by gender (supplementary table S3).

**Table 2:** The association of weekly working hours and unmet medical needs. Bold font indicates statistical significance. Adjusted for age (continuous), gender, household income (quintile), occupation group (blue- or white-collar), smoking status, and shift work (yes or no). [OR=odds ratio; CI=confidence interval.]

Weekly working hours	OR (95% CI)	P-value
Any reason		
<30	1.06 (0.88–1.27)	0.534
30–52	1 (Reference)	
>52	**1.36 (1.22–1.52)**	**<0.001**
Lack of time		
<30	**0.65 (0.48–0.88)**	**0.005**
30–52	1 (Reference)	
>52	**1.64 (1.42–1.90)**	**<0.001**
Economic burden		
<30	**1.61 (1.17–2.21)**	**0.003**
30–52	1 (Reference)	
>52	1.26 (0.99–1.61)	0.058

The association between household income and unmet medical needs is shown in table 3. Compared to the highest household income group, the lowest household income groups showed a significantly higher OR for unmet medical needs at 1.86 (95% CI 1.48–2.34). In the gender-stratified analysis, this association was more prominent among males than females (supplementary table S4). Unmet medical needs due to economic burden were predominantly associated with household income; however, those driven by a lack of time were not significantly associated. The OR for unmet medical needs owing to economic burden in the low household income group was 13.17 (95% CI 8.62–20.12), compared with the highest group.

**Table 3:** The association of household income and unmet medical needs. **Bold font indicates statistical significance.** Adjusted for age (continuous), gender, weekly working hours (<30, 30–52, >52), occupation group (blue- or white-collar), smoking status, and shift work (yes or no). [OR=odds ratio; CI=confidence interval.]

Household income	OR (95% CI)	P-value
Any reason (quintile)		
1^st^	**1.86 (1.48–2.34)**	**<0.001**
2^nd^	**1.28 (1.10–1.49)**	**0.001**
3^rd^	1.08 (0.94–1.24)	0.269
4^th^	1.07 (0.94–1.21)	0.292
5^th^	1 (Reference)	
Lack of time (quintile)		
1^st^	0.74 (0.49–1.11)	0.141
2^nd^	0.90 (0.73–1.11)	0.326
3^rd^	0.94 (0.78–1.13)	0.502
4^th^	1.04 (0.89–1.22)	0.626
5^th^	1 (Reference)	
Economic burden (quintile)		
1^st^	**13.17 (8.62–20.12)**	**<0.001**
2^nd^	**4.92 (3.32–7.28)**	**<0.001**
3^rd^	**3.56 (2.46–5.17)**	**<0.001**
4^th^	**2.31 (1.58–3.36)**	**<0.001**
5^th^	1 (Reference)	

The same analytical method was used to calculate the OR of long working hours by quintiles of household income level (table 4). The OR of the >52 hours per week group was higher than that of the 30–52 hours per week group as the quintile of household income level increased, suggesting a dose–response relationship according to household income level. The adjusted OR of long working hours from low to high quintiles are as follows: 1.28 (95% CI 0.74–2.23), 1.32 (95% CI 1.02–1.71), 1.36 (95% CI 1.10–1.68), 1.38 (95% CI 1.11–1.72), and 1.58 (95% CI 1.28–1.95). In addition, the association between long working hours and unmet medical needs due to lack of time was strongest among the lowest income group (OR 4.26, 95% CI 1.92–9.46).

**Table 4:** The odds ratios (OR) of weekly working hours for unmet medical needs according to household income. Bold font indicates statistical significance. Adjusted for age, gender, occupation group, smoking status, and shift work. [CI=confidence interval]

Household income	Weekly working hours				

	<30		30–52	>52	
		
	OR ^a^ (95% CI)	P-value	OR ^a^ (95% CI)	OR ^a^ (95% CI)	P-value
Any reason (quintile)					
1^st^	1.18 (0.72–1.94)	0.505	1 (Reference)	1.28 (0.74–2.23)	0.378
2^nd^	0.88 (0.60–1.31)	0.545	1 (Reference)	1.32 (1.02–1.71)	0.034
3^rd^	1.19 (0.82–1.72)	0.356	1 (Reference)	1.36 (1.10–1.68)	0.005
4^th^	1.11 (0.78–1.57)	0.565	1 (Reference)	1.38 (1.11–1.72)	0.004
5^th^	0.65 (0.39–1.09)	0.101	1 (Reference)	1.58 (1.28–1.95)	<0.001
Lack of time (quintile)					
1^st^	0.45 (0.10–2.10)	0.308	1 (Reference)	4.26 (1.92–9.46)	<0.001
2^nd^	0.54 (0.26–1.09)	0.085	1 (Reference)	2.01 (1.40–2.89)	<0.001
3^rd^	0.56 (0.30–1.05)	0.072	1 (Reference)	1.42 (1.05–1.91)	0.021
4^th^	0.82 (0.50–1.35)	0.437	1 (Reference)	1.60 (1.22–2.10)	<0.001
5^th^	0.50 (0.24–1.05)	0.067	1 (Reference)	1.72 (1.32–2.24)	<0.001
Economic burden (quintile)					
1^st^	1.08 (0.59–1.97)	0.799	1 (Reference)	0.99 (0.61–1.6)	0.952
2^nd^	2.46 (1.30–4.67)	0.006	1 (Reference)	1.72 (1.14–2.59)	0.009
3^rd^	1.69 (0.84–3.43)	0.144	1 (Reference)	1.91 (1.19–3.08)	0.007
4^th^	1.05 (0.26–4.19)	0.945	1 (Reference)	1.71 (0.85–3.46)	0.135
5^th^	1.08 (0.59–1.97)	0.799	1 (Reference)	0.99 (0.61–1.60)	0.952

a OR for unmet medical needs of the long working hours group (>52 hours/week) compared with the standard working hours group (30–52 hours/week).

**Figure 1:**
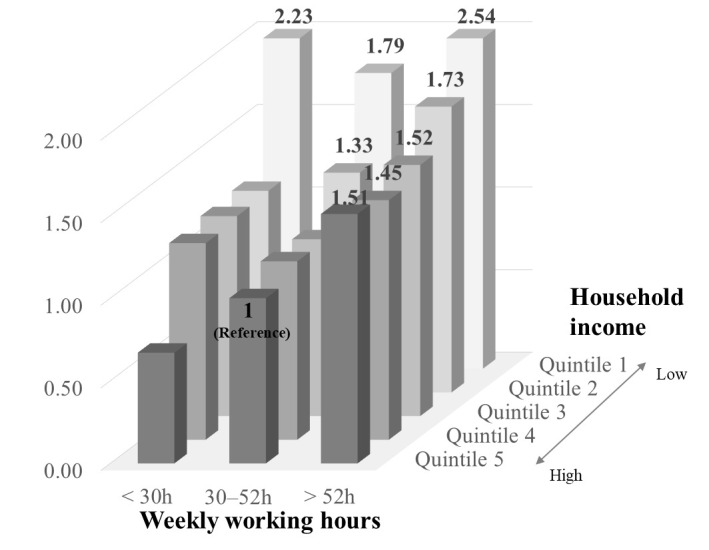
The odds ratios (OR) for unmet medical needs according to household income and weekly working hours. OR were estimated compared with those who worked 30–52 hours per week and were in the high household income group, with adjustment for age, gender, occupation group, smoking status, and shift work. Statistically significant OR are marked above the bars.

Figure 1 shows the combined association between long working hours and quintiles of household income and unmet medical needs. Compared to the reference group (those who worked 30–52 hours per week in the highest household income group), participants with >52 hours per week in the quintiles 5 (highest), 4, 3, 2, and 1 (lowest) had OR for unmet medical needs at 1.51 (95% CI 1.23–1.86), 1.45 (95% CI 1.18–1.80), 1.52 (95% CI 1.24–1.87), 1.73 (95% CI 1.37–2.19), and 2.54 (95% CI 1.59–4.06), respectively. Short-time workers in the lowest income group had significantly high unmet medical needs (OR 2.23, 95% CI 1.42–3.47). However, short-term workers in the highest income group were associated with a lower unmet medical need (OR 0.67, 95% CI 0.40–1.10), although this was not statistically significant.

In the sensitivity analyses with different weekly working hours grouping, the results remained robust (supplementary table S5–7). Long working hours (>52 hours per week) were associated with unmet medical needs, compared with those who worked 30–40 hours a week (OR 1.59, 95% CI 1.40–1.80). The size of the association between long working hours (>52 hours per week) and unmet medical needs was also bigger in high-income groups, as OR (95% CI) of quintiles 1 (lowest), 2, 3, 4, and 5 (highest) were 1.56 (95% CI 0.85–2.88), 1.31 (95% CI 0.98–1.77), 1.70 (95% CI 1.30–2.21), 1.54 (95% CI 1.20–1.98), and 2.04 (95% CI 1.61–2.59), respectively.

## Discussion

The results of our analysis showed that the association between long working hours and the risk of unmet medical needs differed according to household income. In the highest household income, the risk of unmet medical needs was 1.58-fold higher among those who worked >52 hours per week, than those who worked 30–52 hours per week. However, this association was not significant in the lowest income group.

Our finding, of a significant association between long working hours and the risk of unmet medical needs, is in line with those of previous studies. In a study by Ha et al (21), the unmet medical need experience rate of regular workers was found to be 13.3%; it also found that long working hours in Korea create restrictions on the use of medical care by workers because most of them have unmet medical needs owing to a lack of time. Similarly, Seok et al (13) reported that long hours of work might limit healthcare use, thus resulting in unmet medical needs.

Our study found that the effects of long working hours on workers’ health problems could differ according to their economic status. Specifically, there is a significant association between long working hours and an increased risk of unmet medical needs among the high- but not the low-income group. A previous study supported this finding, where the association between long working hours and 10-year risk of cardiovascular disease was significant only among the highest income group among men, while the association between household income and 10-year risk of cardiovascular disease was significant only among the lowest income group (16).

A possible explanation for these findings is that working hours could affect health differently, depending on the environment and reactions of individuals. Differences in the risk of unmet medical needs due to a lack of time across groups with different income levels may reflect the effect of autonomy on the regulation of working time between the groups. The health effects of long working hours are exacerbated by workers’ lack of control over working hours, which is more common in lower-income groups (22). Moreover, a lack of autonomy in the use of time restricts access to healthcare. In situations of mandatory overtime, workers further lose the ability to make time for health management. This lack of control over working hours may also be linked to working time mismatch (ie, the difference between actual hours and preferred hours) that negatively affects workers’ utility (23). According to a study using a periodical survey of Korean workers, poor subjective health status was reported among those whose actual working hours did not correspond to their preferred working hours (24). Another study using a nationally representative longitudinal survey in the United Kingdom also showed that a discrepancy between preferred and actual working hours induces psychological stress (25), which is an important aspect while considering the relationship between long working hours and health problems (26). Additionally, there is a possibility that the level of psychological stress from long working hours differs according to preferred working hours, related to the economic status of individuals. From an economic view, for better health, the marginal utility of an increase in leisure time and the marginal utility of an increase in income resulting from the addition of working hours, can vary depending on income level.

Long working hours can reduce recovery and rest time after work, cause work–life imbalance, and disrupt health behaviors, thereby leading to a deterioration in workers’ health (27–29). Meanwhile, it has been recognized that the higher the wage, the better the worker’s health (30, 31). Nevertheless, the potential difference by income level with regard to the impact of working hours on health has been of limited interest. Workers sometimes change jobs to spend more time with their families, even if it involves a loss of labor income. In contrast, some workers accept jobs with longer working hours if they want higher income at the expense of their own free time (32, 33). While individuals in the high-income group may not want to increase their working hours for more earnings, those in low-income groups may need to increase their income by working longer hours. Considering that workers make choices about working hours based on their income–leisure preferences, the findings of this study could also be interpreted from the perspective of working time preference and mismatch. According to the results of a recent study conducted in Korea, although the negative effects on health generally increase as working hours increase, the health conditions of workers who worked in accordance with their preferred working hours were found to be the best, regardless of working hours (24). Given the backward-bending labor supply curve, unwanted long working hours can adversely affect health in the group whose income is above a certain level; however, the negative health effects of long working hours may not be apparent on the surface in those who want to receive higher wages by working long hours, owing to low income. Therefore, the results of this study support the complex inter-relationship between income, working hours, and health, by considering the concept of a working hours-preference mismatch.

However, the weaker association between long working hours and the risk of unmet medical needs among lower-income groups does not imply that there are no detrimental effects of long working hours among these groups. The association might be obscured by the negative effects of low income. This was supported by the results that, when compared to the highest household income and standard working hour group, long working hours showed a higher OR for unmet medical needs in the lowest income group, than in the highest income group (figure 1). This implies that financial hardship is a more fundamental health hazard than working longer hours among the low-income group. Our data indicated that the low-income group showed the highest prevalence of unmet medical needs, regardless of working hours. Along with the results of table 3, this finding suggests that only when basic economic needs have been met do the adverse effects of prolonged working hours outweigh the positive income effects and harm the health of people with sufficient resources.

This study has several strengths. Specifically, the use of nationally representative panel data allows for the generalization of results to the broader population of South Korea. Panel data also permit us to focus on within-person changes in the experience of unmet medical needs as working hours change, controlling for unobserved time-invariant characteristics. Failure to control for time-invariant individual characteristics could bias the estimation results if there were correlations between the observed covariates and these unobserved factors. This is in marked contrast to the majority of previous studies based on cross-sectional data in this area. Another notable strength is that this is a unique study investigating how this association between long working hours and unmet medical needs differed by household income level. This could help us understand the mechanisms and identify at-risk populations, potentially contributing to the development of improved public health policies.

Despite these strengths, our study has some limitations. First, the experience of unmet medical needs and working hours was defined using self-reported answers from respondents. However, previous studies have indicated that subjective unmet medical needs are as important as clinically evaluated ones because, in many circumstances, people perceive their healthcare needs better than healthcare professionals (34). Some studies using subjective and objective methods tend to show fairly consistent results (35), and consequently, many studies use subjective measures (7, 13, 21). Second, the participants were restricted to South Korea, limiting the generalizability of our findings to other populations with different medical care systems and cultural contexts, which affect the level of unmet healthcare needs. Therefore, our results should be carefully considered when generalizing them to other countries. Third, the reason for unmet medical care needs should be interpreted with caution, as the respondent could choose only one of the most important reasons. Fourth, because our study sample size in the lowest income status was relatively small, it could have led to biased results, derived from selection bias. It would be possible that workers with extremely low resources, including time and health, could not work, and thus were not included in our analysis or – even if they were included – could not work long hours. Therefore, the results should be interpreted cautiously, owing to the problem of uneven distribution according to income group. Finally, because we used existing data which lacked information on time-varying living and working conditions, job stress, work time control, work–family conflicts, and mental disorders, among others, we could not control for these factors in our analysis. This is an area for further research.

### Concluding remarks

This study suggests that the association between long working hours and the risk of unmet medical needs varies based on household income as it was found predominantly among high-income groups, and increases in household income had protective effects on the risk of unmet medical needs. This implies that future policies should consider not only limiting working hours but also compensating workers’ income to adequately protect them from the health risks associated with long working hours. We hope our results will contribute to accumulating evidence in supporting the implementation of effective strategies for protecting workers’ health.

## Supplementary material

Supplementary material
