# Supplementary material for: Differential impact of working hours on unmet medical needs by income level: a longitudinal study of Korean workers
Source: Scand J Work Environ Health. 2022 Feb 25;48(2):109–17. doi: 10.5271/sjweh.3999 (PMC9045236; doi:10.5271/sjweh.3999)
Supplement: Supplementary material [file SJWEH-48-109-S001.pdf]

Differential impact of working hours on unmet medical needs by income level: a longitudinal study of Korean workers[1]

by Dong-Wook Lee, PhD, Jaesung Choi, PhD, Hyoung-Ryoul Kim, PhD, Jun-Pyo Myong, PhD,3 Mo-Yeol Kang, PhD 2

1. *Supplementary material*
2. *Correspondence to: Mo-Yeol, Kang, MD, PhD, Associate Professor, Department of Occupational and Environmental Medicine, Seoul St. Mary's Hospital, College of Medicine, The Catholic University of Korea, 222, Banpo-daero, Seocho-gu, Seoul, 06591, Republic of Korea. [E-mail: snaptoon@naver.com] ORCID: 0000-0002-1682-865X*

Supplementary Figure S1. Depicts of the study population 2

Supplementary Table S1. Demographic characteristics of the included participants and excluded participants 2

Supplementary Table S2. Distribution of unmet medical need and weekly working hours across the study period.. 4

Supplementary Table S3. The association of weekly working hours and unmet medical needs by gender 5

Supplementary Table S4. The association of household income and unmet medical needs. ... 6

Supplementary Table S5. Distribution of unmet medical need and weekly working hours (<30, 30–40, 41–52, and >52) across the study period.. 7

Supplementary Table S6. The association of weekly working hours (<30, 30–40, 41–52, and >52) and unmet medical needs. 8

Supplementary Table S7. The odds ratios of weekly working hours (<30, 30–40, 41–52, and >52) for unmet medical needs according to household income. 9

Supplementary Figure S1. Depicts of the study population

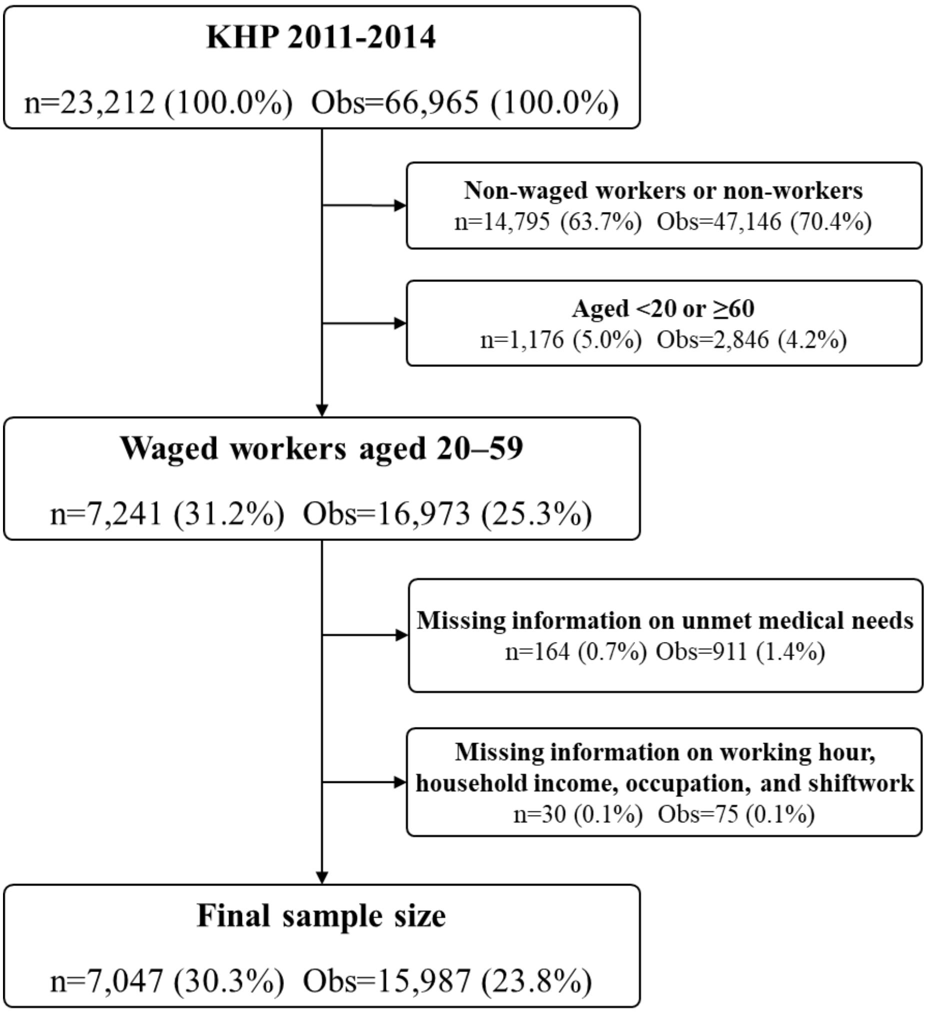

Supplementary Table S1. Demographic characteristics of the included participants and excluded participants

	2011		2012		2013		2014	
	Included n (%)	Excluded n (%)	Included n (%)	Excluded n (%)	Included n (%)	Excluded n (%)	Included n (%)	Excluded n (%)
Gender								
Male	2,216 (55.9)	6,085 (46.6)	2,119 (55.6)	5,590 (46.4)	1,966 (55.4)	5,215 (46.2)	2,590 (55.6)	6696 (46)
Female	1,749 (44.1)	6,985 (53.4)	1,692 (44.4)	6,471 (53.7)	1,585 (44.6)	6,073 (53.8)	2,070 (44.4)	7863 (54)
Age								
<20		4,044 (30.9)		3,585 (29.7)		3,260 (28.9)		4107 (28.2)
20-29	522 (13.2)	1,204 (9.2)	518 (13.6)	1,144 (9.5)	480 (13.5)	987 (8.7)	590 (12.7)	1328 (9.1)
30-39	1,121 (28.3)	1,112 (8.5)	1,027 (27)	909 (7.5)	921 (25.9)	802 (7.1)	1,184 (25.4)	942 (6.5)
40-49	1,402 (35.4)	1,377 (10.5)	1,374 (36.1)	1,239 (10.3)	1,284 (36.2)	1,165 (10.3)	1,664 (35.7)	1471 (10.1)
50-59	920 (23.2)	1,360 (10.4)	892 (23.4)	1,238 (10.3)	866 (24.4)	1,149 (10.2)	1,222 (26.2)	1475 (10.1)
≥60		3,973 (30.4)		3,946 (32.7)		3,925 (34.8)		5236 (36)
Employment status								
Waged workers	3,965 (100)	953 (7.3)	3,811 (100)	855 (7.1)	3,551 (100)	867 (7.7)	4,660 (100)	1157 (8)
Employer or self-employer		2,076 (15.9)		1,943 (16.1)		1,858 (16.5)		2402 (16.5)
Non-waged workers		571 (4.4)		540 (4.5)		507 (4.5)		653 (4.5)
Non-workers		5,118 (39.2)		4,787 (39.7)		4,517 (40)		5935 (40.8)
Missing		1 (0)		11 (0.1)		15 (0.1)		8 (0.1)
Not applicable		4,352 (33.3)		3,925 (32.5)		3,524 (31.2)		4404 (30.3)
Household income[a]								
1st Quintile	144 (3.6)	2,119 (16.2)	136 (3.6)	1,926 (16.0)	129 (3.6)	1,824 (16.2)	137 (2.9)	2,314 (15.9)
2nd Quintile	550 (13.9)	2,625 (20.1)	535 (14.0)	2,475 (20.5)	452 (12.7)	2,311 (20.5)	580 (12.5)	2,972 (20.4)
3rd Quintile	901 (22.7)	2,895 (22.2)	845 (22.2)	2,645 (21.9)	786 (22.1)	2,488 (22.0)	1,056 (22.7)	3,219 (22.1)
4th Quintile	1,109 (28)	2,797 (21.4)	1,052 (27.6)	2,607 (21.6)	1,036 (29.2)	2,406 (21.3)	1,341 (28.8)	3,154 (21.7)
5th Quintile	1,261 (31.8)	2,629 (20.1)	1,243 (32.6)	2,394 (19.9)	1,148 (32.3)	2,251 (19.9)	1,546 (33.2)	2,896 (19.9)
Missing		5 (0)		14 (0.1)		8 (0.1)		4 (0)

a Quintiles of equalised household income among total participants in each year.

Supplementary Table S2. Distribution of unmet medical need and weekly working hours across the study period.

	2011<br>n (%)	2012<br>n (%)	2013<br>n (%)	2014<br>n (%)
Unmet medical need				
<30	39 / 253 (15.4)	52 / 272 (19.1)	43 / 218 (19.7)	37 / 295 (12.5)
30–52	388 / 2662 (14.6)	343 / 2613 (13.1)	399 / 2561 (15.6)	386 / 3430 (11.3)
>52	227 / 1050 (21.6)	166 / 926 (17.9)	141 / 772 (18.3)	141 / 935 (15.1)
Total	654 / 3965 (16.5)	561 / 3811 (14.7)	583 / 3551 (16.4)	564 / 4660 (12.1)
Unmet medical need due to lack of time				
<30	8 / 253 (3.2)	17 / 272 (6.3)	13 / 218 (6.0)	9 / 295 (3.1)
30–52	167 / 2662 (6.3)	167 / 2613 (6.4)	206 / 2561 (8.0)	227 / 3430 (6.6)
>52	115 / 1050 (11.0)	96 / 926 (10.4)	85 / 772 (11.0)	89 / 935 (9.5)
Total	290 / 3965 (7.3)	280 / 3811 (7.3)	304 / 3551 (8.6)	325 / 4660 (7.0)
Unmet medical need due to economic burden				
<30	13 / 253 (5.1)	16 / 272 (5.9)	20 / 218 (9.2)	15 / 295 (5.1)
30–52	77 / 2662 (2.9)	64 / 2613 (2.5)	70 / 2561 (2.7)	42 / 3430 (1.2)
>52	38 / 1050 (3.6)	41 / 926 (4.4)	30 / 772 (3.9)	18 / 935 (1.9)
Total	128 / 3965 (3.2)	121 / 3811 (3.2)	120 / 3551 (3.4)	75 / 4660 (1.6)

Supplementary Table S3. The association of weekly working hours and unmet medical needs by gender

Weekly working hours	Male OR (95% CI)	*p*	Female OR (95% CI)	*p*
Any Reason				
<30	1.23 (0.89 – 1.70)	0.204	0.94 (0.75 – 1.17)	0.574
30–52	1 (Reference)		1 (Reference)	
>52	**1.37 (1.19 – 1.58)**	**<0.001**	**1.46 (1.22 – 1.73)**	**<0.001**
Lack of Time				
<30	0.85 (0.48 – 1.51)	0.584	**0.55 (0.38 – 0.78)**	**<0.001**
30–52	1 (Reference)		1 (Reference)	
>52	**1.69 (1.39 – 2.05)**	**<0.001**	**1.73 (1.39 – 2.16)**	**<0.001**
Economic Burden				
<30	**2.11 (1.23 – 3.62)**	**0.007**	1.32 (0.90 – 1.94)	0.157
30–52	1 (Reference)		1 (Reference)	
>52	**1.41 (1.02 – 1.97)**	**0.040**	1.17 (0.81 – 1.68)	0.413

Adjusted for age (continuous), household income (quintile), occupation group (blue collar or white collar), smoking status, and shiftwork (yes or no)
Bold font indicates statistical significance.

Supplementary Table S4. The association of household income and unmet medical needs.

	Male		Female	
Household income	OR (95% CI)	*p*	OR (95% CI)	*p*
Any Reason				
1st quintile	**2.36 (1.69 – 3.29)**	**<0.001**	**1.49 (1.08 – 2.04)**	**0.014**
2nd quintile	**1.35 (1.09 – 1.67)**	**0.007**	1.22 (0.98 – 1.51)	0.076
3rd quintile	**1.22 (1.001 – 1.48)**	**0.044**	0.95 (0.78 – 1.16)	0.634
4th quintile	1.08 (0.90 – 1.29)	0.424	1.07 (0.90 – 1.28)	0.443
5th quintile	1 (Reference)		1 (Reference)	
Lack of Time				
1st quintile	0.86 (0.49 – 1.50)	0.597	0.63 (0.35 – 1.15)	0.131
2nd quintile	0.96 (0.71 – 1.30)	0.785	0.85 (0.63 – 1.14)	0.277
3rd quintile	0.98 (0.76 – 1.26)	0.872	0.90 (0.70 – 1.17)	0.426
4th quintile	1.02 (0.81 – 1.28)	0.856	1.06 (0.85 – 1.33)	0.601
5th quintile	1 (Reference)		1 (Reference)	
Economic Burden				
1st quintile	**19.53 (10.17 – 37.50)**	**<0.001**	**9.30 (5.33 – 16.23)**	**<0.001**
2nd quintile	**5.74 (3.19 – 10.33)**	**<0.001**	**4.32 (2.55 – 7.31)**	**<0.001**
3rd quintile	**4.77 (2.72 – 8.38)**	**<0.001**	**2.80 (1.72 – 4.56)**	**<0.001**
4th quintile	**2.51 (1.42 – 4.46)**	**0.002**	**2.17 (1.31 – 3.59)**	**0.003**
5th quintile	1 (Reference)		1 (Reference)	

Adjusted for age (continuous), weekly working hours (<30, 30–52, or >52), occupation group (blue collar or white collar), smoking status, and shiftwork (yes or no)
Bold font indicates statistical significance.

Supplementary Table S5. Distribution of unmet medical need and weekly working hours (<30, 30–40, 41–52, and >52) across the study period.

	2011<br>n (%)	2012<br>n (%)	2013<br>n (%)	2014<br>n (%)
Unmet medical need				
<30	39 / 253 (15.4)	52 / 272 (19.1)	43 / 218 (19.7)	37 / 295 (12.5)
30–40	181 / 1349 (13.4)	165 / 1431 (11.5)	191 / 1415 (13.5)	193 / 1939 (10)
41–52	207 / 1313 (15.8)	178 / 1182 (15.1)	208 / 1146 (18.2)	193 / 1491 (12.9)
>52	227 / 1050 (21.6)	166 / 926 (17.9)	141 / 772 (18.3)	141 / 935 (15.1)
Total	654 / 3965 (16.5)	561 / 3811 (14.7)	583 / 3551 (16.4)	564 / 4660 (12.1)
Unmet medical need due to lack of time				
<30	8 / 253 (3.2)	17 / 272 (6.3)	13 / 218 (6.0)	9 / 295 (3.1)
30–40	71 / 1349 (5.3)	73 / 1431 (5.1)	90 / 1415 (6.4)	120 / 1939 (6.2)
41–52	96 / 1313 (7.3)	94 / 1182 (8)	116 / 1146 (10.1)	107 / 1491 (7.2)
>52	115 / 1050 (11.0)	96 / 926 (10.4)	85 / 772 (11.0)	89 / 935 (9.5)
Total	290 / 3965 (7.3)	280 / 3811 (7.3)	304 / 3551 (8.6)	325 / 4660 (7.0)
Unmet medical need due to economic burden				
<30	13 / 253 (5.1)	16 / 272 (5.9)	20 / 218 (9.2)	15 / 295 (5.1)
30–40	40 / 1349 (3.0)	38 / 1431 (2.7)	38 / 1415 (2.7)	17 / 1939 (0.9)
41–52	37 / 1313 (2.8)	26 / 1182 (2.2)	32 / 1146 (2.8)	25 / 1491 (1.7)
>52	38 / 1050 (3.6)	41 / 926 (4.4)	30 / 772 (3.9)	18 / 935 (1.9)
Total	128 / 3965 (3.2)	121 / 3811 (3.2)	120 / 3551 (3.4)	75 / 4660 (1.6)

Supplementary Table S6. The association of weekly working hours (<30, 30–40, 41–52, and >52) and unmet medical needs.

Weekly working hours	OR (95% CI)	*p*
Any Reason		
<30	**1.21 (1.001 – 1.46)**	**0.049**
30–40	1 (Reference)	
41–52	**1.34 (1.19 – 1.50)**	**< 0.001**
>52	**1.59 (1.40 – 1.80)**	**< 0.001**
Lack of Time		
<30	0.77 (0.56 – 1.04)	0.103
30–40	1 (Reference)	
41–52	**1.45 (1.24 – 1.69)**	**<0.001**
>52	**2.00 (1.69 – 2.37)**	**< 0.001**
Economic Burden		
<30	**1.65 (1.19 – 2.30)**	**0.003**
30–40	1 (Reference)	
41–52	1.06 (0.83 – 1.36)	0.642
>52	1.30 (0.99 – 1.70)	0.056

Adjusted for age (continuous), gender, household income (quintile), occupation group (blue collar or white collar), smoking status, and shiftwork (yes or no)
Bold font indicates statistical significance.

Supplementary Table S7. The odds ratios of weekly working hours (<30, 30–40, 41–52, and >52) for unmet medical needs according to household income.

	Weekly working hours						
	<30		30–40	41–52		>52	
**Household income**	**OR (95% CI)**	***p***	**OR (95% CI)**	**OR (95% CI)**	***p***	**OR (95% CI)**	***p***
Any Reason							
1st quintile	1.42 (0.82 – 2.45)	0.210	1 (Reference)	1.52 (0.91 – 2.55)	0.112	1.56 (0.85 – 2.88)	0.152
2nd quintile	0.88 (0.58 – 1.34)	0.552	1 (Reference)	0.99 (0.73 – 1.34)	0.955	1.31 (0.98 – 1.77)	0.073
3rd quintile	1.44 (0.98 – 2.12)	0.066	1 (Reference)	**1.50 (1.15 – 1.95)**	**0.002**	**1.70 (1.30 – 2.21)**	**<0.001**
4th quintile	1.21 (0.85 – 1.74)	0.290	1 (Reference)	**1.24 (1.00 – 1.52)**	**0.049**	**1.54 (1.20 – 1.98)**	**<0.001**
5th quintile	0.81 (0.48 – 1.37)	0.441	1 (Reference)	**1.63 (1.34 – 1.98)**	**<0.001**	**2.04 (1.61 – 2.59)**	**<0.001**
Lack of Time							
1st quintile	0.61 (0.12 – 3.15)	0.558	1 (Reference)	1.91 (0.66 – 5.51)	0.230	**5.93 (2.18 – 16.13)**	**<0.001**
2nd quintile	0.59 (0.28 – 1.24)	0.165	1 (Reference)	1.22 (0.77 – 1.94)	0.386	**2.24 (1.46 – 3.45)**	**<0.001**
3rd quintile	0.69 (0.36 – 1.33)	0.266	1 (Reference)	**1.54 (1.08 – 2.19)**	**0.018**	**1.80 (1.24 – 2.61)**	**0.002**
4th quintile	0.93 (0.56 – 1.55)	0.773	1 (Reference)	**1.32 (1.001 – 1.74)**	**0.048**	**1.85 (1.35 – 2.52)**	**<0.001**
5th quintile	0.64 (0.30 – 1.36)	0.249	1 (Reference)	**1.72 (1.34 – 2.2)**	**<0.001**	**2.30 (1.71 – 3.11)**	**<0.001**
Economic Burden							
1st quintile	1.19 (0.63 – 2.27)	0.587	1 (Reference)	0.92 (0.48 – 1.77)	0.807	0.49 (0.20 – 1.20)	0.119
2nd quintile	1.15 (0.61 – 2.16)	0.670	1 (Reference)	1.14 (0.69 – 1.87)	0.618	1.05 (0.61 – 1.80)	0.860
3rd quintile	**2.68 (1.32 – 5.42)**	**0.006**	1 (Reference)	1.22 (0.72 – 2.07)	0.468	**1.90 (1.15 – 3.13)**	**0.012**
4th quintile	1.78 (0.85 – 3.70)	0.125	1 (Reference)	1.12 (0.63 – 1.96)	0.702	**2.02 (1.18 – 3.46)**	**0.011**
5th quintile	1.22 (0.29 – 5.13)	0.789	1 (Reference)	1.37 (0.63 – 2.97)	0.433	2.01 (0.89 – 4.52)	0.093

OR for unmet medical needs of the long working hours group (>52 hours/week) compared with the standard working hours group (30–52 hours/week)
Adjusted for age, gender, occupation group, smoking status and shiftwork
OR, odds ratio; CI, confidence interval
Bold font indicates statistical significance.
